# Modelling stillbirth mortality reduction with the Lives Saved Tool

**DOI:** 10.1186/s12889-017-4742-5

**Published:** 2017-11-07

**Authors:** Hannah Blencowe, Victoria B. Chou, Joy E. Lawn, Zulfiqar A. Bhutta

**Affiliations:** 1 Maternal Adolescent Reproductive and Child Health (MARCH) Centre, London School of Hygiene & Tropical Medicine, G11, Keppel Street, London, WC1E 7HT UK; 2 Department of International Health, Johns Hopkins Bloomberg School of Public Health, 615 N. Wolfe Street, Room E5518, Baltimore, MD 21205 USA; 3 Centre for Global Child Health, Hospital for Sick Children, Toronto, ON M5G 0A4 Canada

**Keywords:** Stillbirths, Lives saved tool, Mortality modelling

## Abstract

**Background:**

The worldwide burden of stillbirths is large, with an estimated 2.6 million babies stillborn in 2015 including 1.3 million dying during labour. The Every Newborn Action Plan set a stillbirth target of ≤12 per 1000 in all countries by 2030. Planning tools will be essential as countries set policy and plan investment to scale up interventions to meet this target. This paper summarises the approach taken for modelling the impact of scaling-up health interventions on stillbirths in the Lives Saved tool (LiST), and potential future refinements.

**Methods:**

The specific application to stillbirths of the general method for modelling the impact of interventions in LiST is described. The evidence for the effectiveness of potential interventions to reduce stillbirths are reviewed and the assumptions of the affected fraction of stillbirths who could potentially benefit from these interventions are presented. The current assumptions and their effects on stillbirth reduction are described and potential future improvements discussed.

**Results:**

High quality evidence are not available for all parameters in the LiST stillbirth model. Cause-specific mortality data is not available for stillbirths, therefore stillbirths are modelled in LiST using an attributable fraction approach by timing of stillbirths (antepartum/ intrapartum). Of 35 potential interventions to reduce stillbirths identified, eight interventions are currently modelled in LiST. These include childbirth care, induction for prolonged pregnancy, multiple micronutrient and balanced energy supplementation, malaria prevention and detection and management of hypertensive disorders of pregnancy, diabetes and syphilis. For three of the interventions, childbirth care, detection and management of hypertensive disorders of pregnancy, and diabetes the estimate of effectiveness is based on expert opinion through a Delphi process. Only for malaria is coverage information available, with coverage estimated using expert opinion for all other interventions. Going forward, potential improvements identified include improving of effectiveness and coverage estimates for included interventions and addition of further interventions.

**Conclusions:**

Known effective interventions have the potential to reduce stillbirths and can be modelled using the LiST tool. Data for stillbirths are improving. Going forward the LiST tool should seek, where possible, to incorporate these improving data, and to continually be refined to provide an increasingly reliable tool for policy and programming purposes.

**Electronic supplementary material:**

The online version of this article (10.1186/s12889-017-4742-5) contains supplementary material, which is available to authorized users.

## Background

The worldwide burden of stillbirths is large, with an estimated 2.6 million babies stillborn in 2015 including 1.3 million dying during labour [1]. Stillbirths are an important marker both of maternal health, but also of access to high quality care in pregnancy and particularly around the time of birth [1–3]. These two factors provide an explanation for much of the differences observed in stillbirth rates worldwide, and have received much interest in global health [2–5]. However, despite the large burden and these links to maternal health and quality of care, stillbirths are not included in routine global monitoring [1, 6]. Failure to count stillbirths ignores their impact on women, families and society, underestimates the benefits of investments in maternity care, and has led to failure of investment to reduce stillbirth numbers [5, 7, 8].

Some evidence of increased recognition of the importance of stillbirths on a global level has been seen recently with stillbirths included in the 2014 *Every Newborn Action Plan (ENAP)* to end preventable deaths of newborns, mothers and stillbirths, and as a core indicator in the Global Strategy on Women’s Children’s and Adolescent Health [9, 10]. Improved stillbirth data collection will be essential to allow tracking of progress towards the *ENAP* stillbirth target of ≤12 per 1000 by 2030, and Global Strategy tracking of the stillbirth and linked indicators for the Sustainable Development Goals 3.1 and 3.2. However, in addition to these improved data, tools such as the Lives Saved Tool will be essential to help countries set policy and plan investment to scale up interventions to meet the national *ENAP* stillbirth target.

The Lives Saved Tool (LiST) is an evidence-based software tool used to model the impact of scaling-up health interventions aimed to reduce mortality and morbidity in mothers, newborns, and children under five years of age and stillbirths. The framework has continued to evolve since the original *Lancet* 2003 child survival series examined the impact of increasing coverage of proven child health interventions, and the model has expanded to include further interventions and outcomes as the need for such a tool to help guide policy and program planning became evident [11, 12]. LiST has been adopted by diverse stakeholders, and can be integrated into and strengthen existing planning processes.

Stillbirths were added as an outcome to the LiST model in 2011. The 2011 supplement in BMC Public Health published 7 papers on the impact of interventions on the risk of stillbirth for pregnant women [13–19], and an overview of the modelling approach was included in the original *Lancet* Stillbirth Series [20]. The interventions included in these publications describe the scientific underpinning of the LiST-based estimates for stillbirth reduction reported in the *Lancet* Stillbirth Series 2011, *Lancet* Every Newborn Series in 2014 and *Lancet* Ending Preventable Stillbirths Series 2016 [8, 21, 22]. However, the modelling of stillbirths has evolved during this period, in response to the changing evidence landscape with the suite of interventions modelled varying over time. In this paper we present an overview of the currently included interventions, data inputs and assumptions underlying the existing LiST model for the impact of scaling-up health interventions on stillbirths. Where relevant, details about changes which have occurred over time and potential refinements for future versions are presented.

## Methods

### Theoretical approach and basic modelling structure of LiST

The modelling structure and underlying theory of change of the LiST model has been described in detail in previous publications [23]. Briefly, LiST is a linear, mathematical deterministic model with fixed relationships between inputs (coverage of interventions) and outputs (changes in cause-specific mortality or population-level risk factors for mortality such as intrauterine growth restriction (IUGR)) [24]. Its framework is based upon demographic details, cause of death information, coverage indicators and intervention effectiveness estimates (Table 1). It assumes that mortality rates and the cause of death structure will not change dynamically, and that any differences in the outcomes will be attributable to the change in coverage of key interventions.

**Table 1:** Framework of the Lives Saved Tool

Parameter of the Lives Saved Tool	Source of Data
Demography details (e.g. total population, fertility)	Demographic projections produced by the United Nations Population Division or derived from national or subnational demographic estimates
Cause of death information from country-specific WHO profiles or estimated by using local data sources	Country-specific profiles produced by Maternal and Child Epidemiology Estimation (MCEE) group or estimated based upon local data sources
Coverage levels for a variety of key health interventions that affect child and maternal mortality	Nationally representative household surveys, such as Demographic and Health Surveys (DHS) and Multiple Indicator Cluster Surveys (MICS), or local data sources (e.g. annual state surveys or program data)
Health status indicators for a national or subnational setting	Nationally representative household surveys, such as Demographic and Health Surveys (DHS) and Multiple Indicator Cluster Surveys (MICS), or local data sources (e.g. annual state surveys or program data)
Effectiveness estimates for stillbirth, maternal, neonatal, and child interventions	Cochrane reviews, meta-analyses, Delphi studies, and scientific literature

LiST allows users to set up and run multiple scenarios, called projections, in order to estimate the impact of different health intervention packages based upon coverage at the national or subnational (e.g. region, state, or district) level. These projections provide a structured format for program managers or ministry of health personnel to utilize the latest scientific information about the effectiveness of interventions for maternal, neonatal, and child health; combine information about causes of death and current coverage of interventions to inform planning and decision-making, and help prioritize investments and evaluate existing programs. Currently, there are approximately 60 interventions amenable to scale-up in low and middle income settings in the short to medium term that are categorized across the continuum of care which impact mortality and morbidity outcomes for specific population subgroups (e.g. stillbirths, neonatal, child-under 5, or maternal deaths) or risk factors (e.g. stunting and wasting) modelled in LiST.

Work to review and estimate the cause-specific effects for interventions was previously led by the Child Health Epidemiology Reference Group (CHERG) of the World Health Organization (WHO) and UNICEF following established standard practices and guidelines using an adapted GRADE approach [25]. Consistent with this approach, where evidence from trials or observational studies were not available for a given intervention, an expert Delphi process was undertaken to determine an effect estimate. Data from low and middle income settings were used where possible. Baseline coverage data were based on the latest nationally representative household surveys where available and where such data were not available, expert opinion from the neonatal and stillbirth leads (ZB and JEL) was used to estimate coverage. Oversight and recommendations continue to be led by global health experts organized into technical working groups around a specific area of expertise (e.g. nutrition) which meet on an ad hoc basis to highlight areas for future work and refinements to strengthen the model. Implementation of changes and overall development of the LiST model continues to be led and managed by the Institute for International Programs at the Johns Hopkins Bloomberg School of Public Health.

### Modelling stillbirths in LiST

Special considerations exist when modelling stillbirths in LiST compared to other maternal-child outcomes, in large part due to lack of reliable and routinely collected data related to stillbirths in many data platforms. Stillbirths are modelled independently in LiST compared to other maternal-child outcomes. The overall envelope for the estimated total number of stillbirths at ≥1000 g or ≥28 weeks (third trimester or late fetal deaths) in a country are obtained from WHO estimates [26]. However, in contrast to many other maternal-child outcomes, standard definitions of stillbirth are not universally applied across intervention studies. Hence, it is sometimes necessary to assume that the reductions in mortality for specific interventions that have been reported using a wider definition, e.g. fetal deaths ≥22 weeks or even ‘all fetal losses regardless of gestation’, would also apply to stillbirths at ≥28 weeks.

In addition, systematic estimates of cause of death for stillbirths are not available, therefore stillbirths are modelled using a simple classification based upon timing of stillbirth – antepartum (prior to the onset of labour) or intrapartum (fetal death during labour, but prior to full extraction from the mother). Whilst this is commonly used from a programmatic perspective, even the underlying data to produce country-level estimates according to this dichotomy of stillbirth timing are very sparse, with estimates of the proportion of stillbirths that are intrapartum being based upon a regional median approach for most countries [1] .

Therefore, whereas for maternal deaths, and child deaths following a livebirth, estimates of efficacy or effectiveness to reduce cause-specific mortality (rather than overall mortality) can be used, this is not possible for stillbirths. Stillbirths lack cause of death information and therefore, neither estimates for the envelope of the number of stillbirths from a specific cause, or intervention-based cause-specific mortality changes can be estimated.

**Fig. 1:**
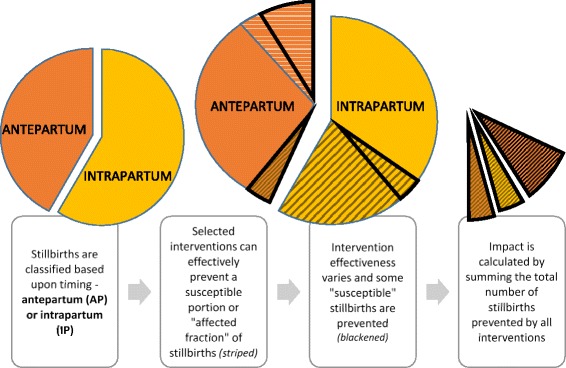
Overview of modelling approach for stillbirths in the Lives Saved Tool

An overview of the modelling approach for stillbirths in LiST is shown in Fig. 1. Any given intervention can have an effect on antepartum or intrapartum stillbirths, or both. The effectiveness of the intervention is derived based on published evidence. Where no published evidence is available for an intervention, the estimate of effectiveness is obtained through expert opinion (see below for specific details). In LiST to calculate the number of stillbirths averted, the estimate of effectiveness is multiplied by the change in coverage. For example, if syphilis detection and treatment is 82% effective in reducing antepartum stillbirths attributable to syphilis, but none of a given population has access to this intervention at baseline, an increase in coverage to 50% is estimated to avert: 82% X (50%–0%) = 41% of all antepartum stillbirths attributable to syphilis.

Some interventions have limited effectiveness so modelled impact is restricted to a sub-group of all deaths. This ‘affected fraction’ is defined in LiST as the proportion of the cause-specific mortality, here antepartum or intrapartum stillbirths, that is considered susceptible to that intervention. Where possible this is calculated as the intrapartum or antepartum stillbirths attributable to the underlying cause in the population based on the prevalence of the risk factor and the increased risk of stillbirth among women exposed to the risk factor (see Additional file 1). If an estimate of increased stillbirth risk is not known for any risk factor, the risk of stillbirth remains the same in those with the exposure as the general population, and hence will underestimate the affected fraction.

For example, balanced energy supplementation has evidence of a 40% reduction in antepartum stillbirths in food-insecure households, but no effect on stillbirths in other households. The number of stillbirths averted is calculated as the estimate of effectiveness multiplied by the coverage multiplied by the ‘affected fraction’. If 20% of a population are food insecure and the coverage was increased from 0 to 50% of food insecure households the estimated number of stillbirths averted would be: 40% X (50%–0%) X 20% = 4% of all antepartum stillbirths.

### Interventions to reduce stillbirths

**Fig. 2:**
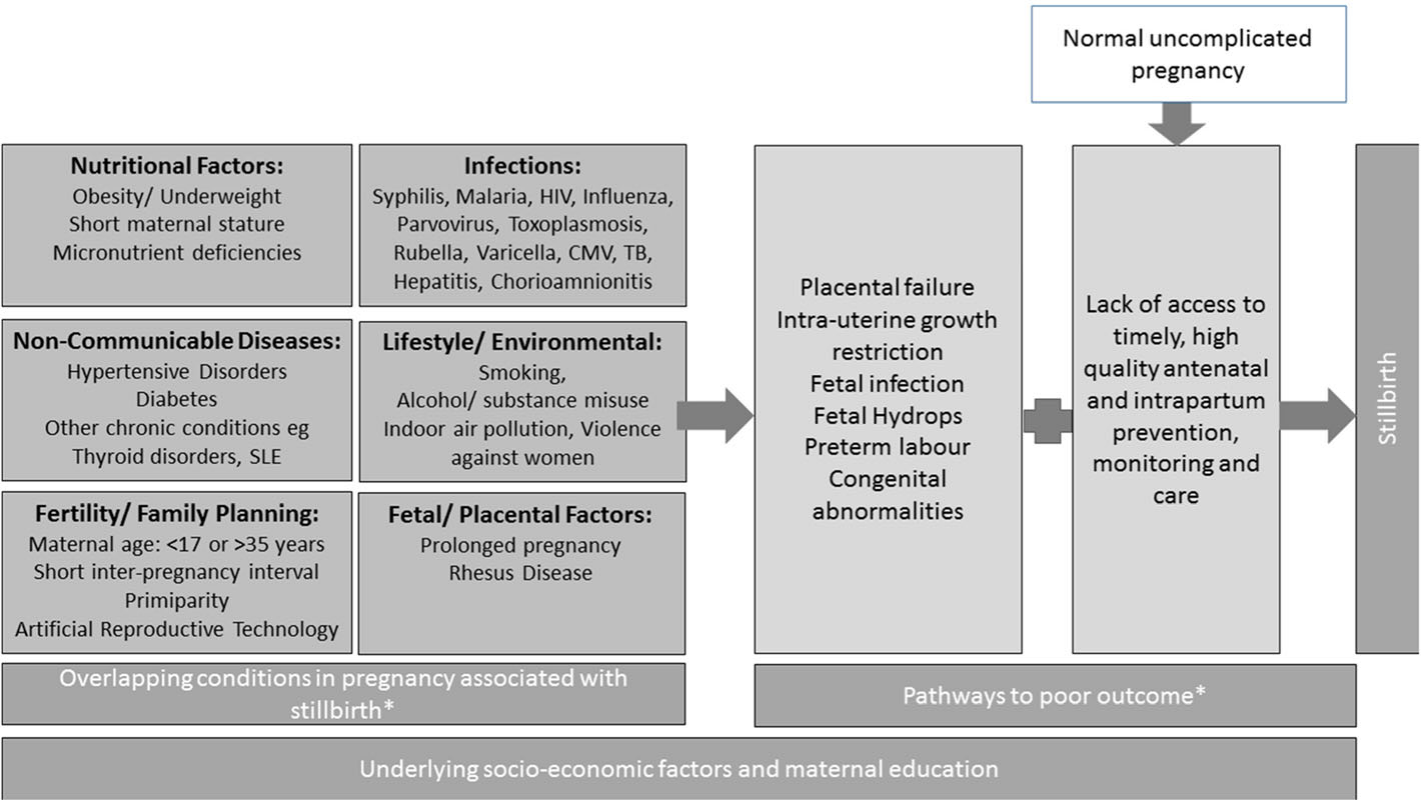
Conceptual framework for known pathways to stillbirth. *This conceptual framework focuses on known conditions in pregnancy associated with stillbirth and understood pathways to stillbirth which are potentially amenable to interventions. The underlying causes and factors in many stillbirths remain unknown, this framework should be revised as further evidence becomes available

**Fig. 3:**
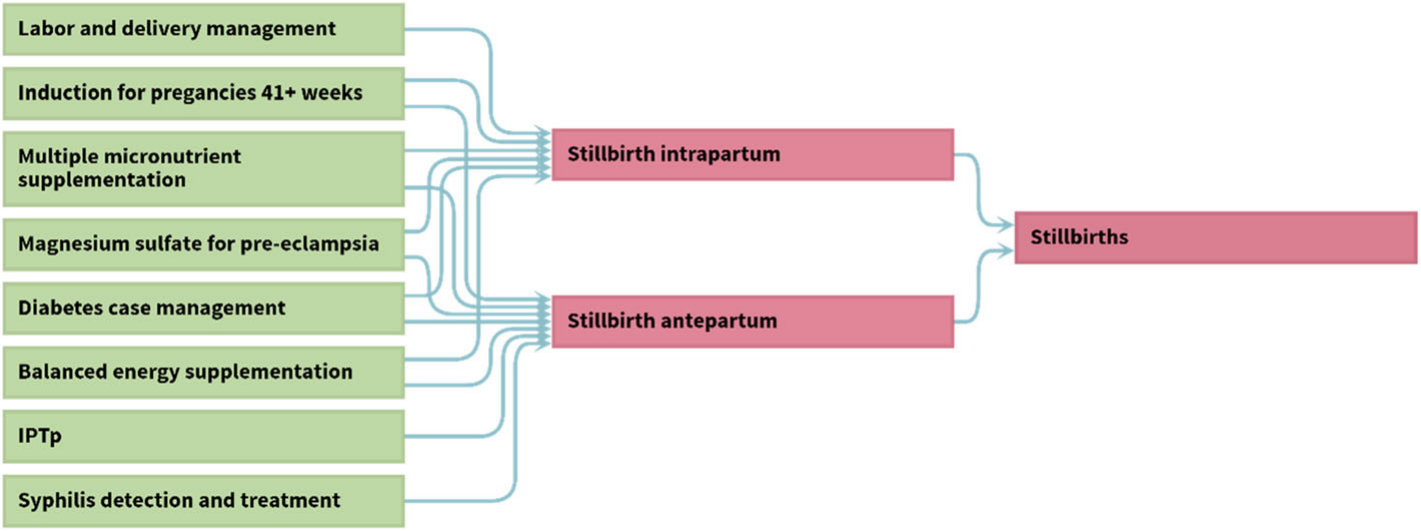
Interventions included in Lives Saved Tool model which impact on stillbirths by timing (antepartum/ intrapartum). This schema represents the LiST modelling of stillbirths as of September 2016

As a first step in defining potential interventions to reduce stillbirths, a conceptual framework of previously identified risk factors for stillbirths can be used (Fig. 2) [1, 27–29]. It should be noted that many of the pathways to stillbirth, particularly antepartum stillbirths, remain inadequately understood, however, for the purposes of this work we are interested in currently modifiable factors. Based on this conceptual framework, a list of potential interventions along the continuum of care from preconception through childbirth care to address these factors, and hence reduce stillbirth mortality, can be considered. Potential interventions of interest were selected on the basis of clear or potential evidence of benefit to reduce stillbirth. Interventions relevant to public health policy, such as those with a large impact on stillbirth reduction that are amenable to scale-up in low and middle income settings in the short to medium term are prioritised. The LiST model currently includes a total of eight interventions that have an impact on stillbirth mortality (Fig. 3). The included interventions have been revised over time (Table 2).

**Table 2:** Summary of interventions effecting stillbirths included in the Lives Saved Tool 2011–2016 ^a^

Period	Intervention	Antepartum Stillbirths	Intrapartum Stillbirths	Current status
*Periconceptual*	*Folic acid supplementation/ Fortification*	*X*	*X*	*Not included. Included in previous versions to Feb 2016*
*Pregnancy*	Multiple micronutrient supplementation	X	X	New intervention added in Feb 2016^b^
*Pregnancy*	Malaria prevention with ITp or ITN^c^	X		Included since 2011
*Pregnancy*	Balanced energy supplementation	X	X	New intervention added in Feb 2016
*Pregnancy*	Syphilis detection and treatment	X		Included since 2011
*Pregnancy*	Diabetes case management	X	X	Included since 2011
*Pregnancy*	Management of hypertensive disorders of pregnancy	X	X	Included since 2011
*Pregnancy*	*Fetal growth restriction detection and management*	*X*	*X*	*Not included. Included in previous versions to May 2015*
*Childbirth*	Labour and delivery management		X	Included since 2011
*Childbirth*	Induction of labour for pregnancies lasting >41 weeks	X	X	Included since 2011

^a^The LiST model continues to evolve, hence some interventions have been added since 2011, and others have been removed as the knowledge base changes. The final column provides details of the current status in version 5.45 (Sept 20 2016)

^b^New evidence from the updated Cochrane suggests that there is no effect of multiple micronutrient supplementation on stillbirths (RR 0.97, 95% CI 0.87–1.09) and this effect and it is likely that the technical working group will recommend that the effect is removed in subsequent LiST revisions

^c^Intervention is labelled as IPTp (Intermittent prophylaxis and treatment of Malaria in pregnancy), or ITN (insecticide treated bednets), although the effectiveness estimate is from ITN alone

## Results

### Summary of included interventions to reduce stillbirth

Of the many possible modifiable underlying causes and risk factors for stillbirth, evidence of scale-able interventions with potential public health effect are only available for a subset. Systematic reviews undertaken prior to the inclusion of stillbirths in LiST in 2011 identified 35 potential interventions, of which 10 were strongly recommended by the technical working group for inclusion in LiST. Since that time two interventions, folic acid supplementation and fetal growth restriction detection and management have been removed, and two new nutritional interventions have been added. Table 2 contains a summary of the interventions modelled within LiST, including the dates that these were included in or removed from the model. Details of the sources of estimates of intervention effects, affected fraction and coverage are provided in Table 3 and Additional file 1, with details described below for each intervention. Further details about the quality of the evidence of effectiveness for the included interventions can be found in Additional file 2. More detailed descriptions of the currently included interventions are provided below:

**Table 3:** Summary of effects, affected fraction and baseline coverage of included interventions for stillbirths in the Lives Saved Tool

Intervention	Effectiveness Estimate^a^ (95% CI)	Source of effectiveness data	Affected fraction^b^	Input data sources for calculating the affected fraction estimate	Source for baseline coverage
Micronutrient supplementation	RR 0.92 (0.86 to 0.99)	Haider et al. [30] 15 RCTs 98,808 women	All stillbirths	-	Zero as default
Malaria prevention with ITp or ITN	RR 0.67 (0.47 to 0.97)	Gamble et al. [45], Ishaque et al [18] 3 cRCTs	Stillbirths attributable to falciparum malaria	Proportion of pregnant women exposed to falciparum malaria [47] Prevalence of placental malaria in those exposed to falciparum malaria (27.8%) [87]^c^ Risk of stillbirth with placental malaria: OR=2.19 (1.49 – 3.22) [88]	Latest DHS/MICS estimate for “% pregnant women receiving 2+ doses of Sp/Fansidar during pregnancy” or “% pregnant women sleeping under an insecticide-treated bednet (ITN)” as a proxy if above NA
Balanced Energy supplementation	RR 0.60 (0.39 to 0.94)	Ota et al. [42] 5 RCTs 3,408 women	Stillbirths occurring in food-insecure households	% pop living <$1.90/day from World Bank [43] is used as a proxy	Zero as default
Syphilis Detection and Treatment	RR 0.18, (0.10 – 0.33)	Blencowe et al. [50] 8 studies 3,931 births	Stillbirths attributable to syphilis	Prevalence data from Newman et al [89] Risk of stillbirth with active syphilis: RR=10.89 (95% CI 6.61 – 17.93) [90]	Defaults based upon ANC4+ coverage^d^: If ANC4+ <40%- assume 20%*ANC4+ If ANC4+ 40-74%-assume 50%*ANC4+ IF ANC4+ 75-95%-assume 70%*ANC4+ If ANC4+>95%-assume 100%*ANC4+
Diabetes screening and management	10% reduction (IQR -5 – 30% for APSB) (IQR 3.5 – 25% for IPSB)	Syed et al. [17] Expert opinion from 31 experts from 6 WHO regions	Stillbirths attributable to diabetes	Prevalence data from International Diabetes Federation Atlas [91] Risk of stillbirth with diabetes: RR=3.38 [92]	Defaults based upon ANC4+ coverage^d^: Assumed to be 5%*ANC4+
Detection and management of hypertensive disease of pregnancy (including treatment with magnesium sulphate)	20% reduction (IQR -10 – 30% for APSB) (IQR 10 – 40% for IPSB)	Jabeen et al. [16] Expert opinion from 33 experts from 6 WHO regions and a range of disciplines	Stillbirths attributable to hypertensive disease of pregnancy	Prevalence data from Dolea et al 2003 [93] Risk of stillbirth with hypertensive disease of pregnancy: RR=2.1^e^	Defaults based upon ANC4+ coverage^d^: Assumed to be 5%*ANC4+
Induction of labour for pregnancies lasting >41 weeks	RR 0.31 (0.12 – 0.88)	Gulmezoglu et al. and Hussain et al. [13, 57] 17 trials 7407 women	Stillbirths attributable to prolonged pregnancy	Prevalence: 7.5% of all pregnancies are estimated to progress post term if no policy to induce at post-term [94] Risk: 1.8 [59]	Default assumption is that 100% of CEmOC deliveries have access to induction of labor for post-term pregnancies, if needed. (Only available for births in CEmOC facilities)
Skilled attendance outside BEmOC or CEmOC facilities	RR 0.77 (0.69 – 0.85)	Yakoob et al. [19] 2 studies	All intrapartum stillbirths	NA	From DHS/ MICS and other nationally representative surveys
Childbirth care in BEmOC facility	45% (IQR 30 – 70%)	Yakoob et al. [19] Expert opinion from 27 experts from 6 WHO regions and a range of disciplines	All intrapartum stillbirths	NA	Defaults based upon facility delivery rates^f^ If Facility delivery: <30% assume 0% BEmOC/ 10% CEmOC 30 – 50% assume 30% BEmOC/ 20% CEmOC 50 – 95% assume 15% BEmOC/ 60% CEmOC >95% assume 0% BEmOC/ 100% CEmOC
Childbirth care in CEmOC facility	75% (IQR 50 – 87%)	Yakoob et al. [19] Expert opinion from 27 experts from 6 WHO regions and a range of disciplines	All intrapartum stillbirths	NA	

^a^ Further details of quality of evidence for estimate of effectiveness are presented in additional file 2

^b^ The affected fraction is the proportion of the time-specific mortality, here antepartum or intrapartum stillbirths, that is considered susceptible to that intervention

^c^ This is based on rates for primigravida in one study. The rate is 20.8% for women in second pregnancy, and 15.6% for higher order pregnancies, and hence may overestimate attributable fraction.

^d^ ANC4+ coverage from household survey data (DHS/MICS). Proportions attending ANC4+ receiving intervention assumptions based on opinion of two experts (Professors Zulfi Bhutta and Joy Lawn)

^e^ Reference not available for source used for approximation of risk. Studies from high income countries suggest aOR 1.3, 1.6 and 2.2 for pregnancy induced hypertension, pre-eclampsia and eclampsia respectively [22]. With similar orders of magnitude in low- and middle-income countries (LMIC) studies [23].

^f^ Facility delivery rates from nationally representative household survey data (DHS/MICS). Proportions receiving BEmOC/ CEmOC assumptions based on expert opinion

### Preventative interventions before and during pregnancy

#### Supplementation with micronutrients before and during pregnancy

Micronutrient deficiencies are common amongst pregnant women and are associated with fetal growth restriction and congenital abnormalities, which can both increase the risk of stillbirth.

Haider et al. found a 9% reduction in all-cause stillbirths with micronutrient supplementation during pregnancy compared to iron with or without folic acid (15 RCTs *n* = 98,808 RR = 0.92, 95% CI 0.86 to 0.99) [30]. Folic acid has previously been shown to have a protective effect to reduce neural tube defects, which are associated with an increased risk of stillbirths, especially when severe [31]. Up until 2016, the effect of folic acid was included in the LiST model for stillbirths (see Additional file 2). This assumed that fortification and supplementation with folic acid were interchangeable. However, whilst the evidence for the effect of folic acid fortification is increasing [32], there is currently no evidence to support an effect of folic acid supplementation, which requires starting peri-conceptually, on all-cause stillbirth mortality (4 trials (*n* = 6597) RR = 1.05 (95% CI 0.54 to 2.05) and therefore the intervention was removed from the LiST model [33] .

No evidence of an effect of Vitamin A, C, D or E, or zinc, magnesium or calcium supplementation on stillbirth reduction has been found in recent Cochrane reviews [34–40], and only the effect for micronutrient supplementation is currently included in the LiST model for stillbirths (see footnote Table 2). The estimate of effect for micronutrient supplementation is for all pregnant women, and hence the affected fraction is 1.0 for both antepartum and intrapartum stillbirths. As coverage of micronutrient supplementation during pregnancy is generally very low, the default for baseline coverage of this intervention in LiST is 0% in all settings (Table 2). It should be noted that in all these micronutrient studies, the number of included cases is relatively low, and as such they may have been under-powered to detect a true effect, especially if the effect size or the affected fraction (on which the intervention may have an effect) is small. However, in these cases, it is less likely that increasing coverage of these interventions at a population level would have a substantial impact on stillbirths in a country.

#### Balanced energy supplementation

Maternal undernutrition is associated with poor fetal growth, low birthweight, and increased risk of stillbirth [41]. Balanced energy supplementation was found to reduce all-cause stillbirths by 40% in food-insecure households (5 RCTs 3408 women. (RR 0.60, 95% CI 0.39 to 0.94)) [42]. The proportion of the population living on <$1.90/day from World Bank is used as the affected fraction, to approximate food-insecure households [43]. It is assumed that this proportion is equal to the proportion of stillbirths occurring in food-insecure households, i.e. that the stillbirth rate in food-insecure is the same as that in insecure households. This is likely to be a conservative estimate, as stillbirth rates may be higher in food-insecure households, where other risk factors for stillbirths are likely to co-exist. It is assumed that the baseline coverage in LiST for this intervention is 0% in all settings.

#### Prevention of malaria in pregnancy

It is estimated that over 125 million pregnant women are at risk of malaria each year, with pregnant women at risk of more severe disease, and their fetus is vulnerable due to effects of maternal systemic febrile illness, impaired placenta function due to placental infection, or the more rare condition of direct fetal infection [44]. Malaria is hence an important cause of stillbirth in countries where malaria is endemic, and is estimated to be attributable for around 20% of stillbirths in sub-Saharan Africa. There is moderate evidence of the effectiveness of insecticide-treated bednets (ITNs) to reduce all-cause fetal loss (stillbirths and miscarriages at all gestational ages) for women during their first and second pregnancies (3 cRCTs (RR 0.67, 95% CI 0.47 to 0.97)) [18, 45]. No significant effect of intermittent preventative treatment in pregnancy (IPTp) to prevent all-cause stillbirths was found in a recent Cochrane review (5 RCTs, 7130 women. RR 1.02, 95% CI 0.76 to 1.36. Moderate quality evidence) [46] .

In LiST, the affected fraction to which this effect estimate is currently applied represents stillbirths attributable to falciparum malaria. Although initially it was planned that impact would be calculated assuming that only women in their 1st or 2nd pregnancies were at increased risk of stillbirth, currently the estimate includes stillbirths attributable to malaria regardless of parity [47] (Table 2). On a country-level, both ITNs and IPTp are used in combination in endemic populations and currently in LiST, the effect of ITNs is also assumed to apply to women using IPTp. Therefore, the baseline coverage for ITNs is estimated from the latest household survey data for “% pregnant women receiving 2+ doses of Sp/Fansidar during pregnancy”, or “% pregnant women sleeping under an insecticide-treated bednet (ITN)” if the former is not available.

### Detection and management of conditions in pregnancy

#### Syphilis case management

In 2012, there were an estimated 930,000 maternal syphilis infections worldwide, leading to 350,000 adverse pregnancy outcomes including 143,000 early fetal deaths and stillbirths [48]. Maternal syphilis infection is able to cross the placenta and infect the fetus in-utero, which can lead to multi-organ damage or congenital developmental abnormalities, hence pregnant women with untreated syphilis infection have 21% higher rates of fetal loss or stillbirth compared to uninfected women [49].

Detection and treatment of syphilis during pregnancy is estimated to reduce all-cause stillbirths by 82% (8 studies, 3931 births. RR 0.18, 95% CI 0.10 to 0.33) [50]. Only stillbirths in women with untreated syphilis could potentially benefit from this intervention. The affected fraction is therefore calculated as the proportion of cases in the whole population that may be attributed to the exposure (untreated syphilis), (see Table 3, Additional file 1). This effect estimate is currently applied only to antepartum stillbirths.

No robust country-level data for the current (baseline) coverage of detection and treatment of syphilis in pregnancy are available for all countries modelled in LiST. Testing and treatment of pregnant women is undertaken during antenatal care visits. As a minimum of two visits is usually required, one to take the blood test and the second to receive the result and get the treatment, it is assumed that a sub-set of those attending full antenatal care (ANC4+) will receive the intervention. Currently, in LiST, it is assumed that a proportion of women attending all four ANC visits in a country will receive testing and treatment for syphilis, and that none receiving fewer than 4 visits will receive such services. These levels were defined by expert opinion, assuming that coverage of the intervention increases with coverage of four ANC visits, for example where ANC4 coverage is low (<40%) only 20% of those attending will receive testing and treatment (Table 3). A limitation of this approach is that the national level ANC4+ coverage is likely to overestimate the coverage in women with syphilis who, due to socio-economic factors, are less likely to access such care than the general pregnant population. Work is currently underway to examine the validity of these coverage proxy assumptions in LiST, and to propose methods to improve these coverage estimates.

#### Diabetes case management

The global epidemic of obesity is affecting pregnancies worldwide [51], with increasing prevalence of type II diabetes in women of reproductive age and gestational diabetes across all regions [52, 53].

Pregnancies affected by gestational diabetes have a higher risk of perinatal mortality than unaffected pregnancies [54], and pregnant women with pre-existing type I or II diabetes are at increased risk of stillbirth even in high income countries (RR 2.9, 95% CI 2.05 to 4.09) [55] .

A review undertaken in 2011 was unable to provide an appropriate effect estimate for the overall package of diabetes detection and management in pregnancy, and hence the effect estimate recommended by the technical working group was based on a Delphi process for expert opinion [17]. This estimated a 10% reduction in diabetic-related stillbirth with diabetes screening and management versus no specific identification or care for women with diabetes [17]. The affected fraction that could potentially benefit from this intervention are stillbirths attributable to diabetes (Table 3). No robust country-level data for the current (baseline) coverage of diabetes screening and management were available. In general, coverage of this intervention is thought to be low and it was therefore assumed, based on expert opinion, that only a small minority (5%) of women accessing 4 or more ANC visits would be receiving this intervention. In practice, this is likely to vary by setting and local policy.

#### Hypertensive disorders of pregnancy case management

Hypertensive disorders of pregnancy, including pregnancy induced hypertension, pre-eclampsia and eclampsia, are common complications which occur and approximately 6% of pregnancies are affected by its more severe forms (e.g., pre-eclampsia and eclampsia) [56]. In high-income countries where early detection is possible, women with either pre-eclampsia or pre-eclampsia have approximately double the risk of stillbirth or neonatal death compared to unaffected women [55]. The risks are likely to be higher in low-resource settings with limited access to detection, and management which may include emergency caesarean section.

The risk is highest for those affected by the more severe forms of hypertensive disorders, and hence currently in LiST the effect of intervention is modelled only for these two more severe conditions.

No suitable evidence was found for a package of interventions for the management of pre-eclampsia and eclampsia, and hence the effect estimate recommended by the technical working group for this intervention was based on a Delphi process for expert opinion. This process suggested a 20% reduction in antepartum and intrapartum stillbirths in women with pre-eclampsia and eclampsia with a package of management interventions including antihypertensive, magnesium sulphate and C-section if needed [16]. The affected fraction is stillbirths attributable to pre-eclampsia or eclampsia (see Table 3). Similar to the case for diabetes, no robust country-level data for the current (baseline) coverage of hypertensive disorders management were available, and coverage of this is thought to be low so it was therefore assumed, based on expert opinion, that only 5% of women accessing 4 or more ANC visits would be receiving this intervention.

There is currently insufficient evidence to estimate the effect of potential interventions including calcium supplementation (see micronutrient interventions above) or aspirin [16, 35] to prevent hypertensive disorders of pregnancy.

#### Prolonged pregnancy case management

Pregnancies lasting longer than around 42 weeks are associated with an increased risk of placental failure and stillbirth. An estimated 14.2% of stillbirths worldwide are attributable to prolonged pregnancy [1]. Induction of pregnancy is an effective method to prevent adverse pregnancy outcomes. A recent Cochrane review was underpowered to detect an effect on stillbirth reduction with induction of pregnancy at 41 completed weeks or more versus expectant management (17 trials, 7407 women. RR 0.30, 95% CI 0.08 to 1.08), however the review found a 69% reduction in perinatal deaths (17 trials, 7407 women. RR 0.31, 95% CI 0.12 to 0.88) [13, 57]. The effect on all cause perinatal mortality is used for the estimate of effectiveness in LiST. This risk data is consistent with population-based data from Denmark, where the risk of fetal death after 42 weeks was reduced by 30–33% (*p* < 0.05) with induction of labour for prolonged pregnancy [58].

The affected fraction that could potentially receive benefit from this intervention are stillbirths attributable to prolonged pregnancy. Gulmezoglu et al. estimate that 7.5% of all pregnancies progress beyond term [57]. The increased risk of stillbirth in post-term pregnancies currently used in LiST is RR 1.8 [59] (Table 2).

Caution however is required prior to consideration of scale-up of this intervention. In the absence of both widespread availability of accurate dating of pregnancies through early ultrasound and safe emergency obstetric care, there is real potential for harm to women and their babies through increased risk of caesarean section and iatrogenic late preterm or early term (37 weeks gestation) birth in cases of mistaken gestational age [60]. For this reason, coverage of this intervention is capped by default to equal coverage or the proportion of births occurring at Comprehensive Obstetric Care (CEmOC)-level facilities.

### Childbirth care

All pregnant women need access to appropriate childbirth care, including skilled birth attendance, and timely access to Basic Emergency Obstetric Care (BEmOC) or Comprehensive Obstetric Care (CEmOC) if complications arise [61]. Higher coverage of birth with a skilled attendant is strongly associated with lower intrapartum stillbirth rates [1, 62]. Similarly, higher coverage of caesarean section up to rates of 10% are associated with sharp reductions in stillbirth rates, although no benefit of higher rates is seen [63].

### Skilled attendance outside BEmOC or CEmOC facilities

All cause stillbirths were estimated to be reduced by 23% (RR 0.77. 95% CI 0.69 to 0.85) for women who delivered at home/outside BEmOC or CemOC facilities with a skilled birth attendant based on a meta-analysis of 2 before and after studies [19]. A similar estimate of 25% reduction was obtained from a Delphi process [19].

### Childbirth care in BEmOC or CEmOC facility

A systematic review of the impact of skilled birth attendance and Emergency Obstetric Care found insufficient evidence from randomised trials or robust quasi-experimental designs to provide an estimate of effect for Emergency Obstetric Care. The estimates of effect for these interventions were therefore obtained through a Delphi expert opinion process [19] which determined that 45 and 75% of intrapartum stillbirths could be prevented by universal access to BEmOC and CEmOC respectively, compared to home delivery without a skilled attendant.

For all of these childbirth care interventions, the affected fraction is all intrapartum stillbirths. The coverage of these three packages of care is interconnected – with the sum of coverage for each of the three packages, plus those delivering at home without a skilled attendant, summing to 100%. The baseline coverage of skilled birth attendance is drawn from data collected during recent household surveys. No suitable data are available to estimate the coverage of emergency obstetric care packages, and hence the baseline estimates of coverage are based upon expert opinion, which assumes a relationship between level of facility delivery and the proportion of women able to access emergency obstetric care packages at those levels of care (Table 2).

## Discussion

In this section we provide details about the limitations of the current approach, alongside details of other potential refinements to strengthen the modelling of the effects of interventions on stillbirths in LiST to further improve its utility to policy makers and program managers.

### Limitations of current approach and recommendations for future improvements

Underlying data to support modelling and estimation for stillbirths are not as strong as for other maternal-child outcomes. Advances have been made in the overall quantity of data to inform country-level estimates of overall stillbirth rates, however as stillbirth and neonatal deaths are modelled independently as separate outcomes within LiST, misclassification between intrapartum stillbirth and early neonatal death, thought to remain common in low-resource settings, may potentially effect the accuracy of the estimates [26]. In addition, the quality of data available to inform the breakdown of stillbirths by timing (antepartum/ intrapartum) remains low [1]. Prevalence data used to calculate the attributable stillbirth mortality currently included are generally based on single time-point data that were available around the year 2010. Since this time, an increase in the measurement and reporting of prevalence data, coupled with improvements in estimation techniques have resulted in improved country-level estimates which could be incorporated into future LiST versions.

The prevalence of specific exposures and stillbirth risk factors may be expected to vary over time in a given country but data are not available to reliably track these trends over time. For most interventions in LiST, these factors are assumed to remain static when estimating maternal, neonatal, or child outcomes with the exception of certain pathogen-related illnesses (e.g. diarrheoa, pneumonia, and meningitis) which shift as certain interventions are scaled up and the etiological burden changes accordingly.

Issues that have been identified with the application of some effect estimates (e.g. folic acid supplementation and fetal growth restriction) have now been addressed. However, data underpinning the effectiveness estimates remains sparse for some interventions. In some cases, effectiveness data must be drawn from trials conducted in high-income countries and in others, where the conduct of randomized controlled trials (RCTs) would not be ethical, estimates are derived from expert opinion.

In addition to this, effectiveness estimates, apart from childbirth care, remain broadly applicable to all stillbirths, and are not time-specific (antepartum/ intrapartum). The current approach assumes that this estimate of affect is equally applicable to all ‘affected’ antepartum stillbirths and intrapartum stillbirths, apart from for syphilis detection and treatment and malaria prevention. However, in practice this is unlikely for many conditions. For example, the effect of micronutrient supplementation is likely to primarily effect antepartum stillbirths. A future approach could consider modelling all stillbirths together and for childbirth care interventions, the percentage of intrapartum stillbirths could be applied as the affected fraction. However, a limitation of this is that the number of averted stillbirths split by timing (antepartum versus intrapartum) would no longer be available to guide policy makers. Further research is required to understand how widespread the use of these data are by programs and policy makers.

Reliable population-based coverage data are not available for many interventions. LiST has therefore relied on expert opinion to estimate likely levels of coverage for interventions using different levels of coverage reported for antenatal and childbirth care as a proxy. The current assumptions create a step-like increase in coverage for some interventions, such as the detection and management of syphilis and childbirth care, whilst very low coverage is assumed for others, such as the detection and management of diabetes and hypertension, even at high ANC4 coverage (see Additional file 3: Figures S1 and S2). New evidence is emerging and may help to inform these coverage assumptions. For example, national estimates of coverage for testing and treatment for syphilis during pregnancy are now available and reveal poor correlation with ANC4 coverage (see Additional file 3: Figure S3) [48]. Work is underway to review currently available evidence to guide these coverage estimates and refine these assumptions in future versions of LiST.

Quantification to estimate the number of lives saved involves some degree of uncertainty. This uncertainty has not been quantified up to now, however initial work has been completed to provide an accompanying range of upper and lower bounds for all model outcomes. In future versions of LiST where these ranges are standard outputs, parameters which would influence the uncertainty ranges for stillbirth outcomes include the effectiveness of stillbirth interventions, stillbirth rates, and the proportion of stillbirths that occur antepartum versus intrapartum. After adequate rounds of beta testing, uncertainty estimates will be available as ranges for stillbirth rates, the number of stillbirths, and the number of stillbirths prevented as estimated by LiST. A full description of the model’s methodology for uncertainty and sensitivity analysis is forthcoming and will be presented in a peer-reviewed publication.

### Updates to effectiveness data for currently reviewed interventions

We undertook a review of the existing literature for the included interventions and searched for any newer evidence published after the previous reviews. No subsequent publications relating the estimate of effect for detection and treatment of syphilis, childbirth care or management of prolonged pregnancy were found.

For detection of fetal growth restriction, a new review found no evidence of benefit for perinatal mortality with symphysis fundal height and serial ultrasound scans compared to clinical palpation alone (1 study. 1639 women. RR 1.25, 95% CI 0.38 to 4.07) [64]. A further new review also found no evidence for biochemical tests of placental function on perinatal mortality (2 trials. 740 women. RR 0.88, 95% CI 0.36 to 2.12) [65]. However, potentially more promising as an intervention to prevent stillbirth is the possible effectiveness of detection and monitoring of women at high-risk of fetal growth restriction [66]. This should be considered for inclusion in future versions of LiST if further evidence applicable to the low and middle income setting becomes available.

There has been a proliferation of studies examining interventions to improve outcomes for diabetes, particularly gestational diabetes, and hypertensive disorders of pregnancy. A full update is now warranted and the previously undertaken systematic reviews on this topic would be bolstered by adding further evidence to support the effect estimate used in LiST [16, 17].

### Evidence to support the addition of other potential interventions to prevent stillbirths

Whilst a plethora of evidence summaries have been published in the last 5 years relating to interventions during pregnancy which may improve stillbirth outcomes, the majority of these studies found no evidence of effect or pertain to interventions which could not be readily scaled-up in low resource settings. In the future it would be desirable to improve the utility of the LiST tool for stillbirths by expanding the tool to include additional interventions targeting major risk factors for or causes of stillbirth. Below we detail some candidate interventions for possible inclusion:

#### Fetal growth restriction case management

Fetal growth restriction, which is frequently due to placental dysfunction, is a common final pathway leading to stillbirth in many cases (Fig. 2). Reliable detection of a fetus whose growth is faltering can allow close monitoring and timely induction of labour or caesarean section prior to fetal death. A package for screening which relies upon body-mass-index (BMI), fundal height and targeted ultrasound scan with appropriate management was estimated by Delphi expert consensus to reduce antepartum and intrapartum stillbirths in high risk pregnancies by 20% [15]. This effect would require the availability of specialised obstetric services including serial ultrasound scans and caesarean section if needed, which are not routinely available in many settings. This effect was included in the LiST model from 2011 to 2016. However, it is currently not included as both the screening package and potential effectiveness in different settings is poorly defined.

#### Nutritional interventions

The last few years have seen the increasing roll out of folic acid fortification to prevent folate-sensitive neural tube disorders [67]. Although previous versions of LiST included an estimate of effect for folic supplementation, research has shown supplementation to be an ineffective strategy, because in most cases, even when taken, supplements are not taken peri-conceptually, and hence started too late to be effective to prevent neural tube defects and associated stillbirths. Folic acid fortification is estimated to reduce primary neural tube defects by 41% (11 studies. RR 0.59, 95% CI 0.52 to 0.68) [14], and assuming fortification does not alter the spectrum of the severity or stillbirth rates amongst affected foetuses, it would be expected that stillbirths attributable to neural-tube defects would also decrease by 41%.

Maternal overweight and obesity are associated with an increased risk of stillbirth, preterm birth, congenital abnormalities, poor fetal growth and low birthweight [55, 68]. Ten percent of stillbirths globally are estimated to be attributable to maternal obesity [1]. To date, there is limited evidence to inform interventions to reduce maternal obesity, either pre-conception or during pregnancy, to improve stillbirth outcomes [69, 70]. This remains an important area for future research to improve the understanding of the biological pathways to increase risk, and which interventions may be amenable to clinical interventions.

#### Fertility/ Family planning interventions

The positive effects of contraception on maternal, newborn and child health have been well-documented [71]. Extremes of maternal age are associated with increased risk of stillbirth. Globally 6.7% of all stillbirths are estimated to be attributable to older maternal age (>35 years) [1]. Adolescent pregnancy is also associated with increased risk, especially for girls under 16 years of age [72], but the magnitude of risk has not yet been quantified as studies frequently include these high risk groups with those 19–20 years who have the lowest biological risk. Short inter-pregnancy intervals are associated with poor perinatal outcomes, however the effect on stillbirths has yet to be quantified [73]. To date, there is insufficient evidence to support the inclusion of family planning interventions to reduce stillbirths in LiST. Currently, modelling stillbirths in LiST does not capture these effects as explained above. Moving forward and as the evidence base increases, inclusion of the potential effects of interventions such as reducing births in girls under 18 years of age, improved inter-pregnancy spacing and reducing unplanned pregnancies would be useful to planners and policy makers as they consider the potential full-impacts of their family planning programs.

#### Environmental and lifestyle interventions

Smoking is associated with an increased risk of stillbirth [74]. Worldwide smoking has been estimated to be attributable for 1.1% of stillbirths worldwide. Unless the trend of increasing rates of smoking among women in low- and middle-income countries (LMICs), especially in South America and parts of Asia, is reversed, smoking will likely become an important cause of preventable stillbirths in these regions [75]. Research has shown that pharmacological and psychosocial interventions can increase smoking cessation in pregnant women, however no effect has been shown on stillbirth rates, and these interventions are expensive and currently remain impractical in most high-burden settings [76, 77].

Household air pollution (HAP) exposure is known to be associated with multiple adverse health outcomes and increased mortality. Exposure is associated with an increased risk of stillbirth [78, 79]. Based on comparing stillbirth risk in observational studies of populations with low versus high HAP exposure, it is estimated that interventions to reduce HAP exposure could reduce stillbirths by 34% (OR 0.66, 95% CI 0.54 to 0.81) [79]. In recent years, multiple studies have assessed interventions to reduce HAP and although the majority have shown effectiveness in reducing HAP levels, the declines have not reached the levels as recommended by the World Health Organization [80].

Violence against women, which remains highly prevalent worldwide, is associated with increased risk of stillbirth [81]. Although 30% of ever-partnered women report having experienced sexual or physical violence, no specific estimates are available for pregnant women [82]. There is some evidence to suggest that advocacy interventions which aim to reduce exposure to violence may produce benefits [83], however, insufficient evidence is available to estimate the effectiveness of such interventions to reduce exposure and stillbirth rates as a result.

#### Prevention of Rhesus disease

Around 1% of stillbirths worldwide are estimated to be attributable to Rhesus (Rh) disease [1]. Antenatal screening for Rh negativity and anti-D immunoglobulin has been highly successful in reducing the incidence of haemolytic disease of the fetus and newborn and reducing stillbirths in countries where it has been implemented [84]. Anti-D has been shown to reduce iso-immunisation [85]. Whilst there is currently insufficient direct evidence to estimate the effectiveness of this intervention on stillbirths [85], the indirect evidence could be considered and may be used to inform future versions of LiST, especially for settings with high prevalence of Rh negativity and adequately strong health systems.

### Detection and management of HIV in pregnancy

A recent review estimated that 0.7% of stillbirths in sub-Saharan Africa may be attributable to HIV infection [1], however the limitations of these data are noted. Roll-out of antiretroviral treatment (ART) to reduce the risk of mother-to-child transmission of HIV infection and the increased uptake of universal provision of maternal ART, in particular, are likely to affect this subgroup of stillbirths. However, as the attributable fraction of stillbirths that would benefit from this intervention is small and data to support the effect of ART on reduction in stillbirth risk are sparse, this intervention is not currently modelled in LiST.

### Modelling using stillbirth cause of death or risk data

As detailed above, no comparable country-level cause-specific stillbirth mortality estimates are currently available for most countries. The World Health Organization has recently published ICD-PM, the application of ICD-10 to perinatal deaths in which it seeks to improve the data availability and comparability for perinatal deaths, including stillbirths, across countries [86]. However, it is likely to be many years before such data are readily available of sufficiently high quality across countries from all regions worldwide to allow global and country-level stillbirth cause-of-death estimates. Even in high-income regions with access and resources to conduct sophisticated investigations, the cause of stillbirth is unknown in around 30% of cases [2]. Hence, updating and extending the approach of using the attributable mortality as the affected fraction to further interventions remains the most feasible approach at present.

## Conclusions

LiST is a widely-used and valuable tool for modelling the impact of scaling-up interventions to reduce stillbirths across high-burden settings. Previous iterations of LiST have been hampered by a lack of robust data for some of the estimates of effectiveness, attributable mortality and coverage. Data for stillbirths remain incomplete but are improving and going forward, the LiST model will continue to evolve to reflect the improving data and expanded evidence base in order to provide an increasingly reliable tool for policy and programming purposes.

## Additional files

Additional file 1: Calculation of attributable stillbirths and affected fraction in the Lives Saved Tool. (DOCX 14 kb)

Additional file 2: Table of quality assessment for effectiveness estimates interventions effecting stillbirths included in the Lives Saved Tool in 2016. (DOCX 27 kb)

Additional file 3: Coverage assumptions for interventions impacting on stillbirths in the Lives Saved Tool. (DOCX 69 kb)
