# Supplementary material for: Modelling stillbirth mortality reduction with the Lives Saved Tool
Source: BMC Public Health. 2017 Nov 7;17(Suppl 4):784. doi: 10.1186/s12889-017-4742-5 (PMC5688483; doi:10.1186/s12889-017-4742-5)
Supplement: Supplementary file 1 — Calculation of attributable stillbirths and affected fraction in the Lives Saved Tool. (DOCX 14 kb) [file 12889_2017_4742_MOESM1_ESM.docx]

Calculation of attributable stillbirths

The calculation of the attributable stillbirths who could benefit from any given intervention is undertaken in two steps:

1. The stillbirth rate in the absence of the risk factor is calculated (the counterfactual – baseline stillbirth rate if this problem did not exist):

$$Baseline SBR=\frac{observed SBR}{\left( Prevalence*risk \right)+\left( 1-Prevalence \right)}$$

2. The stillbirths attributable to the underlying cause or risk factor:

$$Attributable stillbirth rate=Observed SBR-Baseline SBR$$
