# Supplementary material for: Modelling stillbirth mortality reduction with the Lives Saved Tool
Source: BMC Public Health. 2017 Nov 7;17(Suppl 4):784. doi: 10.1186/s12889-017-4742-5 (PMC5688483; doi:10.1186/s12889-017-4742-5)
Supplement: Supplementary file 2 — Table of quality assessment for effectiveness estimates interventions effecting stillbirths included in the Lives Saved Tool in 2016. (DOCX 27 kb) [file 12889_2017_4742_MOESM2_ESM.docx]

Additional File 2: Table of quality assessment for effectiveness estimates interventions effecting stillbirths included in LiST in 2016

| Intervention modelled in LiST | Effect size | Source of estimate of effect | Population receiving intervention included in effect estimate | Intervention included in effect estimate | Comparator population | Outcome included in effect estimate | Quality assessment (GRADE) and notes |
| --- | --- | --- | --- | --- | --- | --- | --- |
| Micronutrient supplementation | RR 0.92  (0.86 to 0.99) | Haider et al[1]  15 RCTs n=98,808 | All pregnant women | Micronutrient supplementation with UNIMMAP or similar (containing one recommended  daily allowance of vitamin A, vitamin B1, vitamin B2,  niacin, vitamin B6, vitamin B12, folic acid, vitamin C, vitamin  D, vitamin E, copper, selenium and iodine with 30 mg of iron  and 15 mg of zinc) | All pregnant women with iron or iron-folic acid supplementation alone | Stillbirths (as reported by trial authors) | High quality evidence |
| Malaria prevention with ITp or ITN | RR 0.67, 95%CI 0.47 to 0.97 | Gamble et al [2], Ishaque et al [3]  3 cRCTs | Pregnant women in their 1^st^ or 2^nd^ pregnancies | Insecticide treated bednets | Pregnant women in their 1st or 2nd pregnancies with no insecticide treated bednets | All fetal losses – stillbirths or miscarriages | High quality evidence for ITN – fetal losses not stillbirth  No strong evidence for IPTp |
| Balanced Energy supplementation | RR 0.60, 95% CI 0.39 to 0.94) | Ota et al [4]  5 RCTs 3,408 women | Nutritionally disadvantaged pregnant women (‘Chronically malnourished’-Gambia, ‘Poor’-India and Bangladesh, ‘low income, black at risk of LBW infant’ New York) and Rural women in Burkina Faso (mean BMI 20.8) | Balanced Energy and protein supplementation (an energy supplement in which  less than 25% of the energy is from protein) | Nutritionally disadvantaged pregnant women receiving no balanced energy supplementation or placebo alone | Stillbirth (death after 20 weeks’ gestation and before birth) | Moderate-quality evidence |
| Syphilis Detection and Treatment | RR 0.18,  (0.10 – 0.33) | Blencowe et al [5]  8 studies, 3,931 births | Women with active syphilis receiving intervention | At least 2.4MU of penicillin | Women with untreated active syphilis | Stillbirths (as reported by study authors) | Low quality evidence from observational studies with insufficient or no control for important confounding variables |
| Diabetes screening and case management | 10% reduction  (IQR -5 – 30% for APSB)  (IQR 3.5 – 25% for IPSB) | Syed et al [6] | Pregnant women with diabetes | Diabetes screening and management | Routine acre with no specific identification or care for women with diabetes | Antepartum stillbirth or Intrapartum stillbirth | Expert opinion from 31 experts from 6 WHO regions |
| Identification and management of hypertensive disease of pregnancy (including treatment with magnesium sulphate) | 20% reduction  (IQR -10 – 30% for APSB)  (IQR 10 – 40% for IPSB) | Jabeen et al [7]  Expert opinion based on DELPHI process | Pregnant women with pre-eclampsia or eclampsia | Package for identification and management of hypertensive disease of pregnancy including: use of an appropriate  antihypertensive, magnesium supplementation in case of preeclampsia/eclampsia and availability of C-section when required. | Routine acre with no specific indentification management of hypertensive disease of pregnancy | Antepartum stillbirth or Intrapartum stillbirth | Expert opinion from 33 experts from 6 WHO regions and a range of disciplines |
| Induction of labour for pregnancies lasting >41 weeks | 69%  (based on effect for perinatal mortality) | Gulmezoglu et al [8]  17 trials  7407 women | Pregnant women prior to 42 weeks gestation, majority from high income settings | Induction of labour prior to 42 weeks | Expectant management | Perinatal mortality | Moderate quality evidence |
| Skilled attendance outside BEmOC or CEmOC facilities | RR 0.77  (0.69 – 0.85) | Yakoob et al [9]  2 studies | Pregnant women in study area | (1) Training of women as village midwives to provide birth attendance.  (2) Training at all levels of health care system | (1) women delivering in control area  (2) control area women delivering in health centre or unsupervised village deliveries | (1) perinatal mortality  (2) Stillbirths | Very low quality evidence |
| Childbirth care in BEmOC facility | 45%  (IQR 30 – 70%) | Yakoob et al [9]  Expert opinion based on DELPHI process | All women in labour | Childbirth care in a BEmOC facility | Non-attended delivery in the community | Intrapartum stillbirths | Expert opinion from 27 experts from 6 WHO regions and a range of disciplines |
| Childbirth care in CEmOC facility | 75%  (IQR 50 – 87%) | Yakoob et al [9]  Expert opinion based on DELPHI process | All women in labour | Childbirth care in a BEmOC facility | Non-attended delivery in the community | Intrapartum stillbirths | Expert opinion from 27 experts from 6 WHO regions and a range of disciplines |

Estimation of effectiveness of folic acid to reduce stillbirths

Up until 2016, folic acid supplementation/ fortification was included in the LiST model for stillbirths with an effect estimate of RR-0.59 (95%CI - 0.52 – 0.68) based on 11 fortification studies for the reduction of primary neural tube defects [10]. It was assumed that the reduction in incidence would approximate the reduction in mortality from neural tube defects. The affected fraction was stillbirths attributable to folic acid deficiency calculated using data on the prevalence of folic acid deficiency and increased risk of stillbirths associated with folic acid deficiency. In view of the lack of availability of coverage data, baseline coverage was set to zero. These previous estimates assumed that the interventions folic acid fortification and supplementation were interchangeable, however, whilst the evidence for the effect of folic acid fortification is increasing [11], no evidence to support an effect of folic acid supplementation on stillbirth mortality has been found [12]. Although the benefit of supplementation is biologically plausible if taken peri-conceptually, in practice, most women initiate supplementation too late in pregnancy (around 6 – 12 weeks) or not at all. Folic acid supplementation has therefore been removed from the LiST model at present.

References:

1. Haider, B.A. and Z.A. Bhutta, *Multiple-micronutrient supplementation for women during pregnancy.* Cochrane Database Syst Rev, 2015(11): p. Cd004905.

2. Gamble, C., J.P. Ekwaru, and F.O. ter Kuile, *Insecticide-treated nets for preventing malaria in pregnancy.* Cochrane Database Syst Rev, 2006(2): p. Cd003755.

3. Ishaque, S., et al., *Effectiveness of interventions to screen and manage infections during pregnancy on reducing stillbirths: a review.* BMC Public Health, 2011. **11 Suppl 3**: p. S3.

4. Ota, E., et al., *Antenatal dietary education and supplementation to increase energy and protein intake.* Cochrane Database Syst Rev, 2015(6): p. Cd000032.

5. Blencowe, H., et al., *Lives Saved Tool supplement detection and treatment of syphilis in pregnancy to reduce syphilis related stillbirths and neonatal mortality.* BMC Public Health, 2011. **11 Suppl 3**: p. S9.

6. Syed, M., et al., *Effect of screening and management of diabetes during pregnancy on stillbirths.* BMC Public Health, 2011. **11 Suppl 3**: p. S2.

7. Jabeen, M., et al., *Impact of interventions to prevent and manage preeclampsia and eclampsia on stillbirths.* BMC Public Health, 2011. **11 Suppl 3**: p. S6.

8. Gulmezoglu, A.M., et al., *Induction of labour for improving birth outcomes for women at or beyond term.* Cochrane Database Syst Rev, 2012. **6**: p. CD004945.

9. Yakoob, M.Y., et al., *The effect of providing skilled birth attendance and emergency obstetric care in preventing stillbirths.* BMC Public Health, 2011. **11 Suppl 3**: p. S7.

10. Imdad, A., M.Y. Yakoob, and Z.A. Bhutta, *The effect of folic acid, protein energy and multiple micronutrient supplements in pregnancy on stillbirths.* BMC Public Health, 2011. **11 Suppl 3**: p. S4.

11. Castillo-Lancellotti, C., J.A. Tur, and R. Uauy, *Impact of folic acid fortification of flour on neural tube defects: a systematic review.* Public Health Nutr, 2013. **16**(5): p. 901-11.

12. De-Regil, L.M., et al., *Effects and safety of periconceptional oral folate supplementation for preventing birth defects.* Cochrane Database Syst Rev, 2015(12): p. Cd007950.
