# Supplementary material for: Modelling stillbirth mortality reduction with the Lives Saved Tool
Source: BMC Public Health. 2017 Nov 7;17(Suppl 4):784. doi: 10.1186/s12889-017-4742-5 (PMC5688483; doi:10.1186/s12889-017-4742-5)
Supplement: Supplementary file 3 — Coverage assumptions for interventions impacting on stillbirths in the Lives Saved Tool. (DOCX 69 kb) [file 12889_2017_4742_MOESM3_ESM.docx]

Additional File 3: Coverage assumptions for interventions impacting on stillbirths in LiST:

Figure 1 - Coverage of antenatal care interventions, by access to antenatal care

*Notes – Current assumptions assume that coverage of diabetes and hypertension detection and management are the same in a given setting, and remain very low, even at full coverage of antenatal care. Coverage of syphilis detection and treatment is assumed to increase in a step-wise manner, with near-universal coverage once antenatal care coverage reaches 100%.*

Figure 2 - Coverage of levels of childbirth care amongst facility births

*Notes – up to 30% facility birth rate in a population 90% of these births are assumed to occur in facilities with no access to BEmOC or CEmOC. Large steps in different proportions of facility births having access to the different levels of care are seen – especially between 29% coverage to 30% coverage and 49% coverage to 50% coverage.*

Figure 3 – Correlation of estimates of coverage of ANC4 and test and treat for syphilis in pregnancy

*Data source – Wijesooriya 2016 [1] : Current national estimates of coverage of test and treat for syphilis are poorly correlated with ANC4 coverage*

References:

1. Wijesooriya, N.S., et al., *Global burden of maternal and congenital syphilis in 2008 and 2012: a health systems modelling study.* Lancet Glob Health, 2016. **4**(8): p. e525-33.
